# Feature Extraction and Classification of EHG between Pregnancy and Labour Group Using Hilbert-Huang Transform and Extreme Learning Machine

**DOI:** 10.1155/2017/7949507

**Published:** 2017-02-19

**Authors:** Lili Chen, Yaru Hao

**Affiliations:** ^1^School of Mechatronics & Vehicle Engineering, Chongqing Jiaotong University, Chongqing 400074, China; ^2^School of Chongqing Key Laboratory of Urban Rail Transit Vehicle System Integration and Control, Chongqing Jiaotong University, Chongqing 400074, China

## Abstract

Preterm birth (PTB) is the leading cause of perinatal mortality and long-term morbidity, which results in significant health and economic problems. The early detection of PTB has great significance for its prevention. The electrohysterogram (EHG) related to uterine contraction is a noninvasive, real-time, and automatic novel technology which can be used to detect, diagnose, or predict PTB. This paper presents a method for feature extraction and classification of EHG between pregnancy and labour group, based on Hilbert-Huang transform (HHT) and extreme learning machine (ELM). For each sample, each channel was decomposed into a set of intrinsic mode functions (IMFs) using empirical mode decomposition (EMD). Then, the Hilbert transform was applied to IMF to obtain analytic function. The maximum amplitude of analytic function was extracted as feature. The identification model was constructed based on ELM. Experimental results reveal that the best classification performance of the proposed method can reach an accuracy of 88.00%, a sensitivity of 91.30%, and a specificity of 85.19%. The area under receiver operating characteristic (ROC) curve is 0.88. Finally, experimental results indicate that the method developed in this work could be effective in the classification of EHG between pregnancy and labour group.

## 1. Introduction

Preterm birth is described as the live delivery of babies who occur before 37 weeks of gestational age, which is the leading cause of morbidity and mortality of those babies [1]. More than half of long-term morbidity and 75% of perinatal mortality are related to it [2]. According to the survey of World Health Organization, every year, about 15 million babies are born too early, while the complications of PTB lead indirectly to the death of over 1 million children [3]. PTB has been regarded as an important public health issue for its major contribution on the newborn [4]. In addition, preterm births also have a significant adverse effect on families, the economy, and society [5]. Therefore, good and reliable diagnosis methods are really needed for the detection and prevention of PTB [6].

For the prediction and prevention of PTB in symptomatic women, Sotiriadis et al. [7] carried out transvaginal ultrasound measurement of the cervical length. Hudić et al. [8] analyzed the maternal serum concentration of progesterone-induced blocking factor. Abbott et al. [9] adopted a quantitative method of cervicovaginal fluid fetal fibronectin. Riboni et al. [10] evaluated the efficacy of the phosphorylated insulin-like growth factor-binding protein and of the fetal fibronectin test in predicting preterm delivery. For the prediction of PTB in asymptomatic high-risk women, Bolt et al. [11] explored the combined use of fetal fibronectin testing and transvaginal ultrasound measurement of cervical length. Compared to traditional methods, electrohysterogram (EHG) method is a noninvasive, low-cost, real-time, and effective technique for detecting PTB. Studies have shown that the analysis of EHG can conduct an independent and easier way to monitor labour evolution [12, 13]. Maner et al. [14] in their work determined whether delivery can be predicted using EHG. Moslem et al. [15] in their work investigated whether pregnancy can be successfully monitored and labour can be accurately predicted using EHG.

Constructing effective feature vectors that can best characterize different EHG is a very important step for automatically and effectively identifying labour and pregnancy EHG. In order to diagnose labour, Marque and Duchene [16] derived synchronization from the multichannel abdominal EHG recordings. Toward the goal of detecting preterm birth by characterizing events in EHG, Khalil and Duchêne [17] proposed a method of detection and classification of events in EHG based on dynamic cumulative sum combined with multiscale decomposition. With the end goal to detect preterm deliveries, Moslem et al. [18] analyzed the complexity of EHG by using sample entropy algorithm. Diab et al. [19] used nonlinear methods including time reversibility, sample entropy, Lyapunov exponents, and delay vector variance to distinguish pregnancy and labour EHG. Fergus et al. [20] extracted several time domain and frequency domain features from the EHG recordings to detect PTB. Maner et al. [14] adopted power spectrum frequency analysis to predict term and preterm delivery. Fele-Žorž et al. [21] applied linear and nonlinear techniques to EHG recordings to identify term and preterm deliveries. In order to classify labour contractions and pregnancy contractions in EHG signals, Alamedine et al. [22] chose power spectral density and wavelet packet decomposition to extract features. Diab et al. [23] chose AR model and wavelet decomposition to extract feature. Being composed of a huge amount of intricately interconnected cells and influenced by other physiological signals often makes uterus display strong nonlinearity and nonstationarity [21]. Therefore, nonlinear and nonstationary analysis methods could better extract features to characterize EHG recordings. Recently, researchers have proposed various signal analyzing and processing methods for studying nonlinear and nonstationary physiological signals [24–27]. Compared with the method of HHT, other nonlinear and nonstationary methods including short time Fourier transform, wavelet analysis, and Wigner-Ville distribution are all based on Fourier theory which cannot appropriately reveal the amplitude contribution from each frequency value and have not provided a new definition of frequency mathematically [28]. As a well-known time-frequency analysis method, HHT which is based on EMD makes creative improvement in revealing information of signals in both time and frequency domain [29, 30]. Within nonlinear and nonstationary methods, HHT has been widely used in many researches of physiological signals [31–36].

After extracting feature vectors effectively, it is important to establish reliable identification model for identifying labour and pregnancy EHG recordings. Recently, some machine learning algorithms, such as artificial neural networks (ANNs), multiple linear regression methods, support vector machine (SVM), Bayesian classifier, and ELM have been widely used in binary classification problems. Among these techniques, ANNs which can perform calculations at a very high speed and can adopt itself to learn like humans are the most frequently used classifier for pattern recognition [37]. However, traditional ANNs, such as backpropagation algorithm, can easily get into local optima and the learning speed is slow. SVM, another widely used identification model, which is based on statistical learning theory of Vapnik–Chervonenkis dimension theory and structural risk minimization principle, has strong generalization ability [38]. However, when the training sample size is huge, the training speed is also too slow to meet the real-time requirement [39]. Compared to other learning algorithms, ELM algorithm which is developed on the basis of single-hidden layer feedforward neural network not only avoids falling into local optima but also accelerates the running speed of the identification model [40]. The published literatures have shown that ELM method has been widely used in biomedical signal processing, such as seizure detection in electroencephalogram signals [39, 41].

This paper proposed a novel method for identifying pregnancy EHG recordings and labour EHG recordings based on HHT and ELM. In Section 2, the EHG dataset used in this work and the methods of HHT and ELM are described in detail. The evaluation procedure, the obtained experimental results, and discussion are presented in Section 3. Finally, some conclusions and future work are given in Section 4.

## 2. Materials and Methods

### 2.1. Data Description

**Figure 1:**
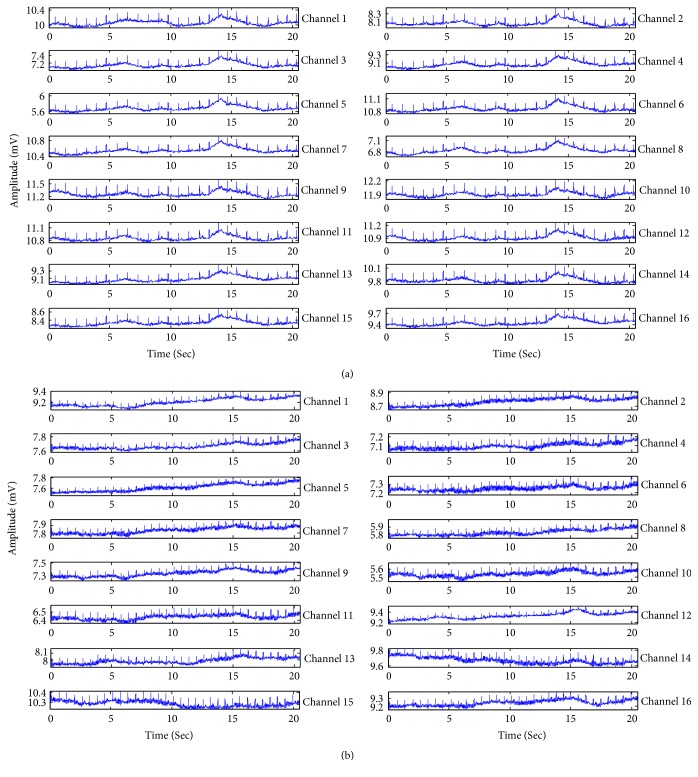
Samples of raw EHG segment. (a) Labour EHG segment and (b) pregnancy EHG segment.

The EHG signals used in this research derived from Icelandic 16-electrode Electrohysterogram Database of PhysioNet [42, 43]. This database comprises 122 EHG recordings (112 pregnancy recordings and 10 labour recordings) made on 45 pregnant women. Pregnancy recordings were made on participants in the third trimester and not suspected to be in labour. Labour recordings were made on participants suspected to deliver within 24 hours. Pregnancy recordings were made at the Akureyri Primary Care Centre in Iceland and the Landspitali University Hospital in Iceland. Labour recordings were made at the Landspitali University Hospital in Iceland and the Akureyri Hospital in Iceland. The protocol was approved by the National Bioethics Committee in Iceland (VSN 02-006-V4). The measurements were performed using a grid of 16 electrodes, arranged in a 4-by-4 matrix positioned on the abdomen. The recording device has an antialiasing filter with a high cut-off frequency of 100 Hz. The signal was sampled at 200 Hz and digitized to 16 bits. The pregnancy recordings duration ranged from 19 to 86 minutes and the labour recordings duration ranged from 8 to 64 minutes. Tocographic signal was simultaneously recorded with the 16-channel EHG recordings. The simultaneous tocodynamometer paper trace provided a reference for the segmentation of the bursts. Actually, the recording times on the tocograph may be up to ±30 seconds from the actual recording or event times.  Figure 1(a) describes a raw labour EHG segment.  Figure 1(b) describes a raw pregnancy EHG segment.

### 2.2. Hilbert-Huang Transform

The HHT technology is composed of two components: EMD and Hilbert spectral analysis. The EMD method is used to decompose a signal into a finite number of intrinsic mode functions (IMFs) which are band-limited [30]. Each IMF is limited to two basic conditions: (1) in the whole data series, the number of extreme points and the number of zero crossings must either be equal or differ at most by one; (2) at any time point, the mean value of the envelopes defined by local maxima and local minima is zero [44].

The EMD can decompose a signal *x*(*t*) into *n* IMFs: *c*_1_(*t*), *c*_2_(*t*),…, *c*_*n*_(*t*) and a residue signal *r*_*n*_(*t*). After EMD, the original signal *x*(*t*) can be reconstructed as follows:

(1) $$\begin{array}{c} x\left(\;t\;\right)=\overset{n}{\underset{i=1}{\sum }}c_{i}\left(\;t\;\right)+r_{n}\left(\;t\;\right). \end{array}$$

In formula (1), *n* is the number of intrinsic modes; *c*_*i*_(*t*) is the *i*th IMF; *r*_*n*_(*t*) is the final residual. Detailed descriptions of EMD algorithm for decomposing *x*(*t*) can be found in [30].

For any real IMF *c*(*t*), its Hilbert transform *c*_*H*_(*t*) is defined as

(2) $$\begin{array}{c} c_{H}\left(\;t\;\right)=\frac{1}{\pi }P\overset{+\infty }{\underset{-\infty }{\int }}\frac{c\left(\tau \right)}{t-\tau }d\tau . \end{array}$$

In formula (2), *P* is the Cauchy principal value of the singular integral. Then, the analytic function *z*(*t*) can be constructed as follows:

(3) $$\begin{array}{c} z\left(\;t\;\right)=c\left(\;t\;\right)+jc_{H}\left(\;t\;\right)=a\left(\;t\;\right)e^{j\phi \left(t\right)}. \end{array}$$

The instantaneous amplitude *a*(*t*) and instantaneous phase *ϕ*(*t*) are defined as

(4) at=ct2+cHt2,ϕt=arctancHtct.

The instantaneous frequency can then be defined as

(5) $$\begin{array}{c} w\left(\;t\;\right)=\frac{d\phi \left(t\right)}{dt}. \end{array}$$

### 2.3. Extreme Learning Machine

The ELM is a new learning algorithm which is developed on the basis of single-hidden layer feedforward neural network (SLFN) [45]. For traditional feedforward neural networks, all the parameters of the neural networks such as input weights and hidden neurons' biases need to be adjusted mostly by the gradient-descent based learning algorithms, which is time-consuming [46]. However, with inappropriate learning steps, gradient-descent-based algorithms are generally inefficient and they tend to fall into local optima. With input weights and hidden layer biases being assigned randomly, a single-hidden neural network with *N* hidden neurons can also achieve any small training error by learning *N* different samples, which has been shown by researches [47, 48]. Thus, only the parameters of output weights need to be adjusted, and the performance of the SLFN would not be influenced by the values of the input weights and hidden layer biases [48]. By assigning the input weights and hidden layer biases randomly and determining the output weights by means of a Moore-Penrose generalized inverse operation of the weight matrices in hidden layers, ELM achieves much faster learning speed and much better generalization ability than traditional learning algorithms [45]. More detailed descriptions of ELM can be found in [49].

The main steps of ELM algorithm are outlined as follows.

**Step 1 .**

Assign arbitrary value for input weights and hidden layer biases.

**Step 2 .**

Select an infinitely differentiable function as the activation function of hidden layer neurons. Then, calculate hidden layer output matrix *H*.

**Step 3 .**

Calculate output weights $\hat{\beta }$.

## 3. Results and Discussion

**Figure 2:**
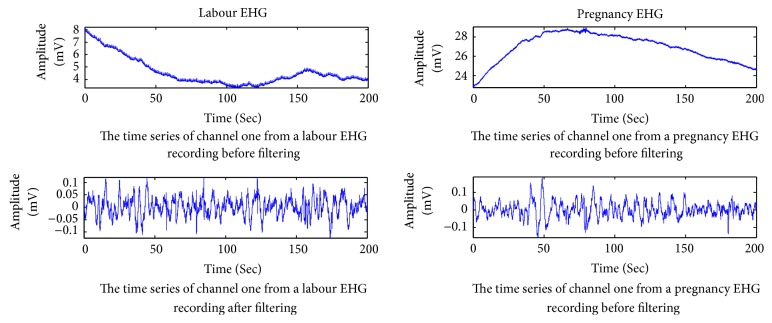
The denoising result of a labour EHG channel and a pregnancy EHG channel.

**Figure 3:**
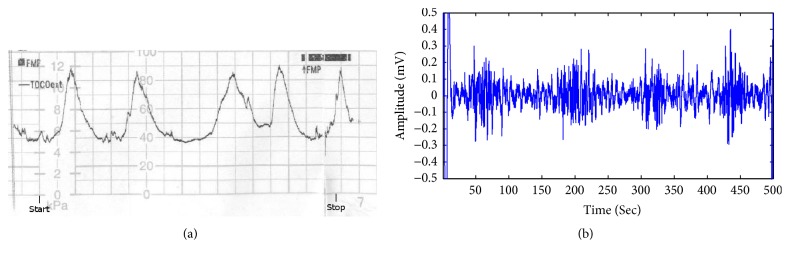
(a) Uterine contraction tracing obtained by tocographic measurement (each small square represents 30 seconds). (b) The channel one of an EHG recording simultaneously recorded when filtered in the 0.1–3 Hz bandwidth.

EHG is a nonstationary signal which contains not only the useful information related to uterine contractile activity but also some unuseful parts such as maternal electrocardiogram, movement artifacts, and baseline. The main frequency band of EHG is known to range between 0.1 and 3 Hz [50]. Therefore, the raw EHG recordings cannot be used directly. Before applying the feature extraction method, three steps are performed in this work: (1) in order to get rid of high frequency noise, movement artifacts, and baseline, all raw EHG recordings were bandpass filtered (0.1–3 Hz) using a six-order Butterworth digital filter; (2) based on the tocographic signal, the bursts of all 122 EHG recordings corresponding to contraction were manually segmented; (3) from these bursts, 150 labour EHG samples and 150 pregnancy EHG samples were selected. Each EHG sample comprises 16 channels with each channel having 4096 points. In addition, there is no overlap between two EHG samples.  Figure 2 illustrates the denoising result of a labour EHG channel and a pregnancy EHG channel. Owing to the limitation of the space, this paper only shows the filtering effects of a labour EHG channel and a pregnancy EHG channel.  Figure 3 describes a tocographic signal and its related preprocessed EHG.

**Figure 4:**
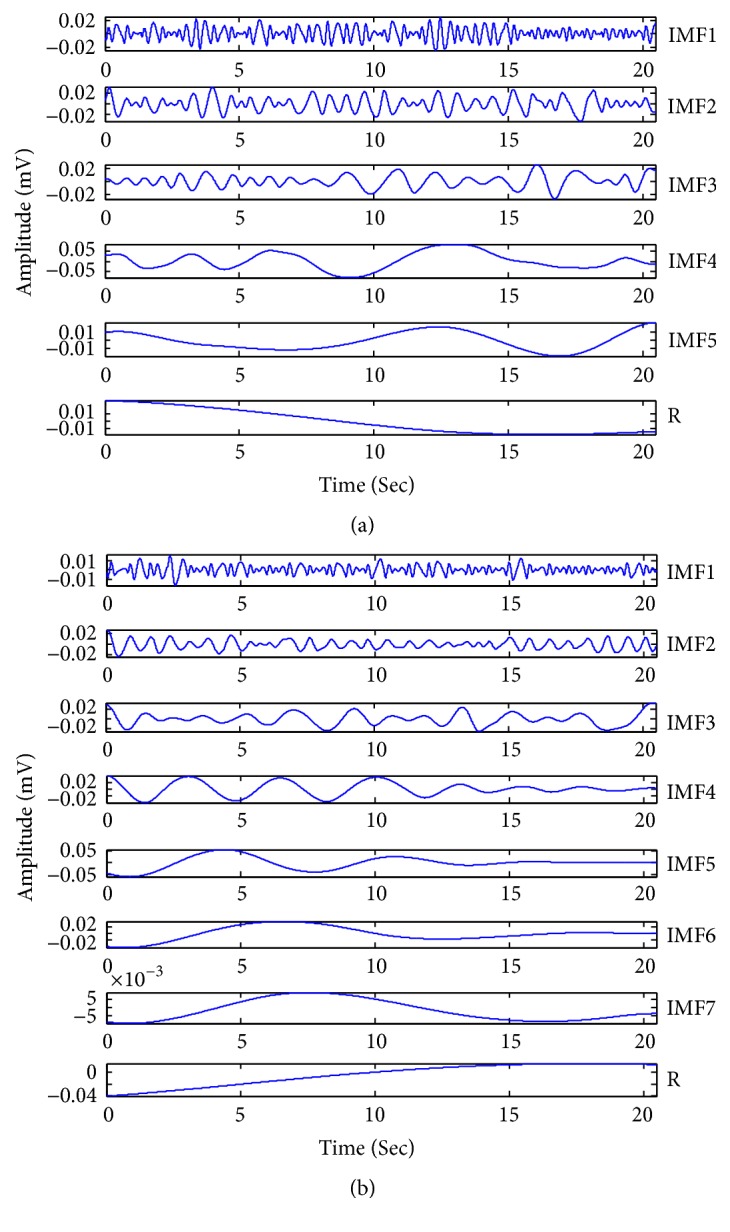
The results of decomposition performed by EMD. (a) The results of decomposition performed by EMD for a labour EHG channel. (b) The results of decomposition performed by EMD for a pregnancy EHG channel.

After all EHG samples were collected, the HHT technology is applied to the EHG samples. The EMD method was firstly used to decompose each channel into a set of IMFs.  Figure 4 describes the results of decomposition performed by EMD of a labour EHG channel and a pregnancy EHG channel. For 150 labour EHG samples and 150 pregnancy EHG samples, it is verified that the lowest number of IMFs in all channels is three. So, feature extraction is based on IMF1, IMF2, and IMF3. For IMF1, IMF2, and IMF3, the methods of feature extraction are the same. Due to the limitation of the space, the experiment process implemented on IMF1 was described in detail in this work.

### 3.1. Feature Extracting Based on IMF1

**Figure 5:**
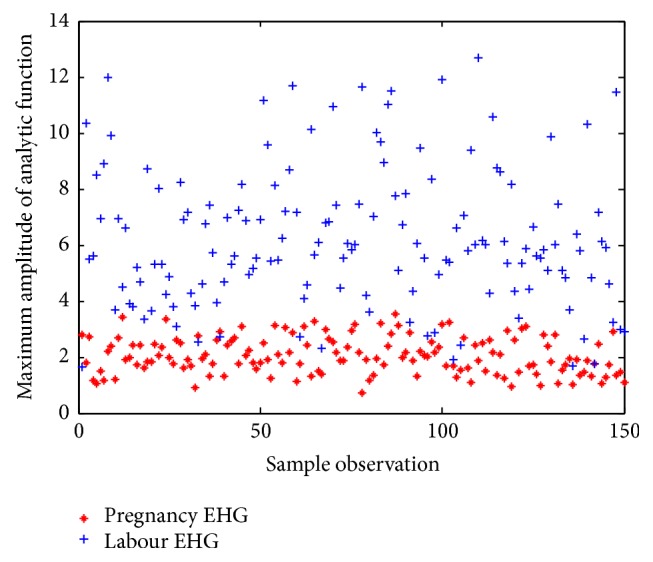
The feature values extracted from IMF1 of channel 1 over all the 150 labour EHG samples and 150 pregnancy EHG samples.

**Figure 6:**
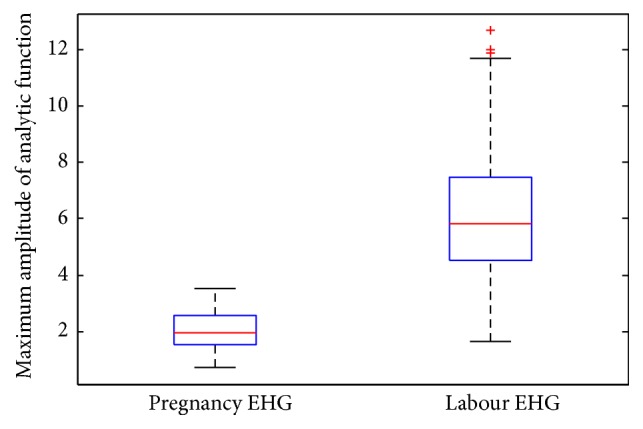
The box plot about maximum amplitudes of analytic function from IMF1 of channel 1 over all 150 labour EHG samples and 150 pregnancy EHG samples.

Hilbert transform was applied to the IMF1 to obtain analytic function. The maximum amplitude of analytic function was extracted as feature. Thus, each sample was characterized by a 16-dimensional feature vector (16 channels).  Figure 5 describes the feature values extracted from IMF1 of channel 1 over all the 150 labour EHG samples and 150 pregnancy EHG samples. From Figure 5, it can be observed that the feature values about labour EHG samples are obviously higher than those of pregnancy EHG samples.  Figure 6 shows the box plot about feature values from IMF1 of channel 1 over all 150 labour and 150 pregnancy EHG samples. In Figure 6, the top and the bottom of each rectangular box stand for the 25th and 75th percentile, respectively, with the median presented inside the box [51]. As one can easily see, there is a significant difference of the feature values between labour EHG samples and pregnancy EHG samples.

### 3.2. ELM Classification Results

**Figure 7:**
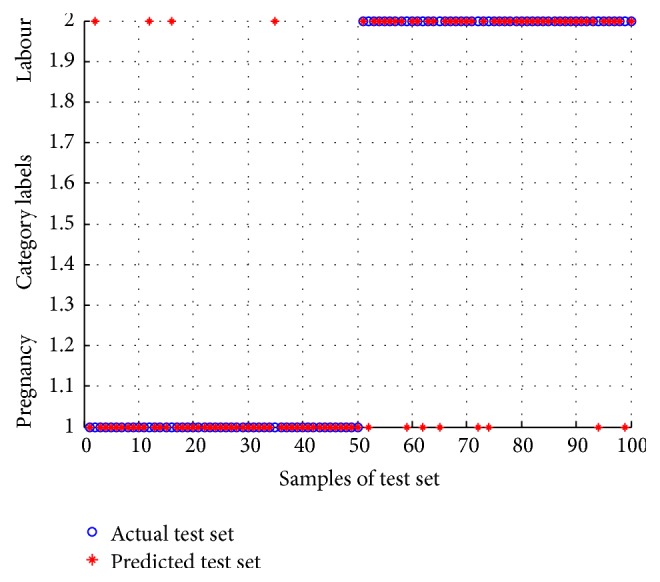
The identification result of test set for IMF1 by using the obtained model.

In this paper, the identification model is constructed based on ELM algorithm. The detailed steps of modeling process are as follows: the number of neurons was set to be 20; the Sigmoidal function was adopted as transfer function; 100 labour feature vectors and 100 pregnancy feature vectors were randomly selected as training set and the remaining 50 labour feature vectors and 50 pregnancy feature vectors were used as test set; construct the labels of training set and test set, where the labels to pregnancy EHG samples were assigned as 1 and labels to labour EHG samples were assigned as 2; enter the input vector of training set and its corresponding labels to the identification model. Then, use the model obtained to predict category labels of test set. The identification results of test set for IMF1 are described in Figure 7. It is clear that when the test set was taken to verify the identification effect of the model, eight labour EHG samples and four pregnancy EHG samples were misjudged.

The identification performance of the ELM classifier can be evaluated by three indexes, sensitivity, specificity, and accuracy, which are defined as follows:

(6) Sensitivity=TPTP+FN,Specificity=TNTN+FP,Accuracy=TP+TNTP+TN+FP+FN.

In formula (6), TP is the total number of labour EHG samples correctly identified; FP is the total number of labour EHG samples incorrectly identified; TN is the total number of pregnancy EHG samples correctly identified; FN is the total number of pregnancy EHG samples incorrectly identified.

Table 1 describes the values of three indexes for IMF1, IMF2, and IMF3. It can be seen that the best classification performance for ELM classifier can be obtained with IMF1 with accuracy of 88.00%, sensitivity of 91.30%, and specificity of 85.19%.

**Figure 8:**
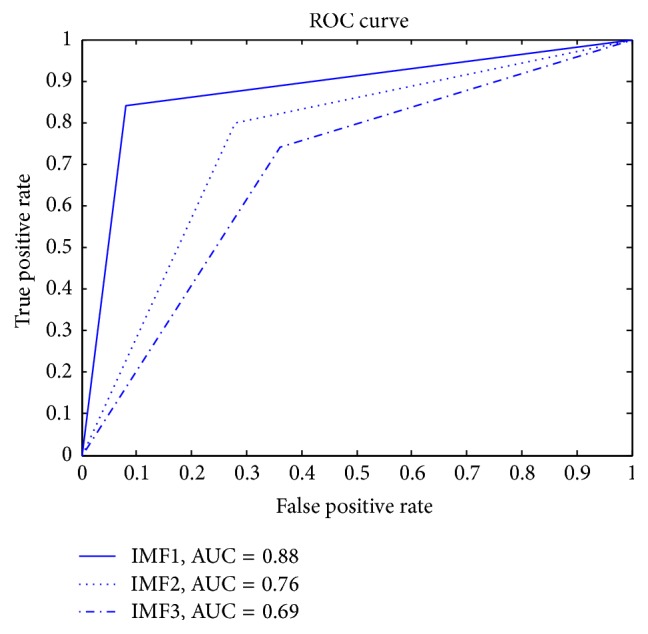
The ROC curve of ELM classifier.

The receiver operating characteristics (ROC) curve shows the trade-offs between sensitivity and specificity, which can intuitively view the entire spectrum of sensitivities and specificities [30]. The area under ROC curve provides a measure of performance for the classification. The larger the area, the better the classification performance [52].  Figure 8 describes the ROC curve of ELM for IMF1, IMF2, and IMF3. As can be seen, the area under ROC curve is 0.88 for IMF1, 0.76 for IMF2, and 0.69 for IMF3.

## 4. Conclusion

The detection of PTB in EHG recordings has great clinical significance. As the detection and prediction of preterm labour from EHG signals are a tedious and time-consuming process, the automatic identification of labour EHG signals has important significance in clinic. Taking into account the nonlinear and nonstationary characteristics of EHG recordings, nonlinear and nonstationary methods may better analyze EHG recordings than traditional linear methods. With the high time-frequency resolution, HHT technology can better reveal the inner scales of signals. Thus, this paper designed a feature extraction method for labour and pregnancy EHG recordings based on HHT and constructed a classification model for labour and pregnancy EHG recordings based on ELM. The experiment results indicate that the features of IMF1 with ELM can provide higher classification of labour and pregnancy EHG. Finally, the main research for future work may include application of the proposed method in this work for identifying other physiological signals.

## Figures and Tables

**Table 1:** Identification performance with three indexes for IMF1, IMF2, and IMF3.

IMF	Number of training data	Number of testing data	Sensitivity (%)	Specificity (%)	Accuracy (%)
IMF1	200	100	91.30	85.19	88.00
IMF2	200	100	74.07	78.26	76.00
IMF3	200	100	67.27	71.11	69.00
